# FXPOI: Pattern of AGG Interruptions Does not Show an Association With Age at Amenorrhea Among Women With a Premutation

**DOI:** 10.3389/fgene.2018.00292

**Published:** 2018-08-03

**Authors:** Emily G. Allen, Anne Glicksman, Nicole Tortora, Krista Charen, Weiya He, Ashima Amin, Heather Hipp, Lisa Shubeck, Sarah L. Nolin, Stephanie L. Sherman

**Affiliations:** ^1^Department of Human Genetics, Emory University School of Medicine, Atlanta, GA, United States; ^2^New York State Institute for Basic Research in Developmental Disabilities, New York, NY, United States; ^3^Department of Gynecology and Obstetrics, Emory University School of Medicine, Atlanta, GA, United States

**Keywords:** FXPOI, POF, FMR1, Fragile X, premutation, menopause, infertility

## Abstract

Fragile X-associated primary ovarian insufficiency (FXPOI) occurs in about 20% of women who carry a premutation allele (55–200 CGG repeats). These women develop hypergonadotropic hypogonadism and have secondary amenorrhea before age 40. A non-linear association with repeat size and risk for FXPOI has been seen in multiple studies women with a premutation: those with a mid-range of repeats are at highest risk (∼70–100 CGG repeats). Importantly, not all carriers with 70–100 repeats experience FXPOI. We investigated whether AGG interruptions, adjusted for repeat size, impacted age at secondary amenorrhea. We have reproductive history information and AGG interruption data on 262 premutation women: 164 had an established age at amenorrhea (AAA) (for some, age at onset of FXPOI) or menopause, 16 had a surgery involving the reproductive system such as a hysterectomy, and 82 women were still cycling at the last interview. Reproductive status was determined using self-report reproductive questionnaires and interviews with a reproductive endocrinologist. For each of these 262 women, *FMR1* repeat size and number of AGG interruptions were determined. We confirmed the association of repeat size with AAA or menopause among women with a premutation. As expected, both premutation repeat size and the quadratic form of repeat size (i.e., squared term) were significant in a survival analysis model predicting AAA (*p* < 0.0001 for both variables). When number of AGG interruptions was added to the model, this variable was not significant (*p* = 0.59). Finally, we used a regression model based on the 164 women with established AAA to estimate the proportion of variance in AAA explained by repeat size and its squared term. Both terms were again highly significant (*p* < 0.0001 for both), but together only explained 13% of the variation in AAA. The non-linear association between AAA and *FMR1* repeat size has been described in several studies. We have determined that AGG interruption pattern does not contribute to this association. Because only 13% of the variation is described using repeat size, it is clear that further research of FXPOI is needed to identify other factors that affect the risk for FXPOI.

## Introduction

The CGG repeat in the 5′ untranslated region of the *FMR1* gene is responsible for three major clinical phenotypes: fragile X syndrome (FXS), fragile X-associated tremor/ataxia syndrome (FXTAS), and fragile X-associated primary ovarian insufficiency (FXPOI). The full mutation form of the *FMR1* gene consists of more than 200 repeats and is abnormally hypermethylated. As a result, mRNA and the gene product, FMRP, are absent, causing FXS (Ashley et al., 1993). Both FXTAS and FXPOI are associated with the premutation form of the *FMR1* gene: an allele with 55–200 CGG repeats (Cronister et al., 1991; Hagerman and Hagerman, 2004). The primary features of FXTAS are intention tremor and gait ataxia, with associated features of parkinsonism, neuropsychological dysfunction, autonomic dysfunction, and peripheral neuropathy (Jacquemont et al., 2003). The lifetime prevalence for FXTAS among men is about 40% and among women is 6–18% (Berry-Kravis et al., 2007; Hagerman and Hagerman, 2016). FXPOI occurs in approximately 20% of women who carry a premutation; these women develop hypergonadotropic hypogonadism and have absent or very irregular cycles prior to the age of 40 (Sherman, 2000). Women with FXPOI have a high risk of infertility and the effects of a hypoestrogenism, including hot flashes, night sweats, and increased risk for osteoporosis. The repeat length of *FMR1* is associated with FXPOI in a non-linear fashion: those with about 70 to 100 CGG repeats are at highest risk, not those with >100 repeats (Sullivan et al., 2005; Ennis et al., 2006; Allen et al., 2007; Tejada et al., 2008; Spath et al., 2011; Mailick et al., 2014).

The molecular mechanism causing FXPOI is unknown. Various potential mechanisms have been investigated in humans and model systems. More definitive answers identified in FXTAS suggest that the secondary hairpin structure resulting from the long repeat in the *FMR1* mRNA is involved in the etiology (for review, see Berman et al., 2014). Researchers have investigated whether these mechanisms can be extended to explain FXPOI as well (for review, see Sherman et al., 2014). In the normal population, the CGG repeat is interrupted by AGG trinucleotides, typically at positions 10 and 20. The length of the CGG repeat and the AGG interruption pattern within that repeat is known to play a role in risk for instability during inheritance (Eichler et al., 1994). With the development of newer technologies to define the repeat structure, larger cohort studies have refined this association with instability (Nolin et al., 2013, 2015; Yrigollen et al., 2013). AGG interruptions are known to disrupt or de-stabilize the hairpin structures and therefore may be protective against clinical outcomes (Napierala et al., 2005).

Because risk of FXPOI does not show a linear relationship with repeat size, as is seen in FXTAS, there are hints that the mechanism may be different from FXTAS. Other molecular consequences of the long premutation repeats may also affect penetrance of the premutation-associated disorders. Carriers of premutation alleles have increased levels of *FMR1* mRNA as CGG repeat size increases and normal to slightly decreased FMRP levels (Tassone et al., 2000). This relationship is seen in male and female premutation carriers; although, not surprisingly, female premutation carriers show greater variation due to the presence of two X alleles and X-inactivation (Allen et al., 2004).

Recently, Lekovich et al. (2017) examined whether repeat size and AGG interruption pattern were associated with three markers of ovarian reserve among women with a premutation: anti-Müllerian hormone (AMH), antral follicle count, and number of oocytes retrieved with *in vitro* fertilization (IVF). Using their three markers, they found that premutation carriers with 70–90 repeats showed significantly lower ovarian reserve than did carriers with fewer or more repeats, consistent with previous studies. They tested the hypothesis that AGGs may be protective on a subset of patients and identified a possible association for higher ovarian reserve among women with two AGGs (Lekovich et al., 2017).

In the current work, we investigate whether the number of AGGs within the CGG repeat of *FMR1* is predictive of age at amenorrhea (AAA) among women with a premutation. This was defined as secondary amenorrhea of at least 4 months and self-report of menopausal levels of follicle-stimulating hormone (FSH) (De Vos et al., 2010). We use this term, AAA, to encompass both the AAA, age at menopause, and onset of FXPOI. In the study of Hipp et al. (2016) on the diagnostic experiences of women with a premutation, they revealed that it took up to 12 years for some women to receive a diagnosis of FXPOI. Delay in diagnosis can cause psychological distress and medical issues due to a prolonged hypo-estrogenic state, such as osteoporosis, dyspareunia due to vaginal atrophy, and cardiovascular disease. Having a simple molecular test to identify premutation alleles with the highest risks for FXPOI has the potential of reducing the time for diagnosis for these women. To this end, we determined the number of AGG interruptions for 262 total women with a premutation. A subset of the women had an established AAA (*N* = 164). The remaining women were still cycling at last interview (*N* = 82) or had undergone a surgical procedure that prevented a determination of AAA (*N* = 16). Regression models and survival analyses were used to determine whether the number of AGGs was significantly associated with AAA in a model that adjusted for CGG repeat size.

## Materials and Methods

### Study Population

The study population was ascertained as previously published (Sullivan et al., 2005; Allen et al., 2007). Briefly, most women were ascertained through families with FXS or a history of FXPOI. A small subset of the women (*n* = 16) were ascertained through a general population survey in the metropolitan Atlanta area (Allen et al., 2005). All women completed a reproductive history questionnaire (Sullivan et al., 2005) and for some, an in-depth interview was conducted by a reproductive endocrinologist (*n* = 83) (Hipp et al., 2016). The reproductive history questionnaire (administered in person, over the telephone, through the mail, or online) included demographic information, age of last menstrual period, and information regarding use of hormone medications. In-depth interviews were conducted over the telephone. During the interviews, questions were asked regarding reproductive status (menopausal or still cycling), other potential causes for amenorrhea (e.g., polycystic ovarian syndrome, exposure to chemotherapy, pregnancy, and breastfeeding), hormone use, and, if applicable, their diagnosis of FXPOI by prior health professionals by laboratory values (FSH) and menstrual history. For many women, the questionnaires and interviews were administered at multiple time points. Research personnel curated all of these data to determine correct assignments for study variables.

The protocols and consent forms for enrollment were approved by the Institutional Review Board at Emory University. Written and informed consent was obtained on all participants.

### Data Collection

Age at amenorrhea was defined as secondary amenorrhea of at least 4 months duration and/or a self-reported menopausal level of FSH (De Vos et al., 2010). Diagnosis was based on self-reporting. If AAA from the in-depth interview differed from information provided on the reproductive questionnaire, the data from the in-depth interview were used. The interviewer (HSH) had the information from all previous reproductive questionnaires available during the interview. Thus, she was able to identify any conflicts and obtain the necessary information to determine the most accurate AAA. Women whose periods stopped because of pregnancy, chemotherapy or radiation, eating disorders, or hormone use were not included in the analyses presented here.

### Laboratory Methods

DNA was extracted from buccal, saliva, or blood samples using Qiagen QiAmp DNA Blood Mini Kit, Gentra Puregene extraction kit, or prepIT-L2P protocol from Oragene. *FMR1* CGG repeat sizes were determined by a fluorescent sequencing method, as described elsewhere (Meadows et al., 1996), using the ABI Prism 377 DNA Sequencer or the ABI 3130XL (Applied Biosystems). For females with only one allele visible, a second PCR-based, hybridization technique was used to identify other possible alleles. The protocol is a modified version of that developed by Brown et al. (1993).

The AGG interruption pattern within the *FMR1* repeat was determined using Xpansion Interpreter^®^ at Asuragen (Austin, TX, United States) or Amplidex PCR/CE *FMR1*^®^ (Asuragen) at the New York Institute for Basic Research in Developmental Disabilities.

### Statistical Analysis

We calculated descriptive statistics for age, race, and body mass index (BMI). Age and BMI are reported as the mean ± SD and as a range. Differences in demographics among premutation groups were examined using an ANOVA model. Race was presented as the percent who self-reported as white. Differences for race were tested for using a χ^2^ analysis. Because there were no significant differences between repeat size groups for age, race, or BMI, further models were not adjusted for these variables. We used linear regression analysis and survival analysis to analyze the association of repeat length and AGG interruption pattern on AAA among women with a premutation. In addition, we confirmed our findings using generalized estimating equations (GEE) and frailty analysis to adjust for the dependency of related individuals. For the linear regression, AAA was used as the outcome variable. For the survival analysis, the “event” was defined as experiencing amenorrhea. For women who had not reached amenorrhea, they were censored using the following criteria: for women still cycling but not taking hormone replacement, they were censored at their age of interview; for women still cycling but taking hormone replacement, they were censored at the last age when they were not taking any hormones; and for women who had a surgery preventing an assignment of AAA, they were censored at their age of surgery.

To be consistent with our previous work, we categorized premutation repeat sizes into three groups: low premutation group was defined as 55–79 repeats, mid-range premutation group was defined as 80–100 repeats, and high premutation group was defined as >100 repeats (Allen et al., 2007). Repeat size groups were only used to investigate differences in demographic variables; in all regression and survival models, repeat size was used as a continuous variable.

The exact position of AGG interruptions was not known for all samples (only number of AGGs was available on 74 of the 262 samples). To estimate the approximate number of 3′ pure repeats, we used the following calculation: for 0 AGG interruptions, 3′ pure repeats were equal to repeat size; for 1 AGG interruption, 3′ pure repeats were equal to repeat size minus 10; and for 2 AGG interruptions, 3′ pure repeats were equal to repeat size minus 20. We tested the accuracy of our assumptions using the data on the 188 samples with known positions. Only five did not agree with our assumptions within one CGG repeat: these five samples had one AGG interruption and 5, 5, 12, 13, and 19 CGG repeats before the interruption (**Supplementary Table S1**).

## Results

Women with a premutation were included in this analysis if reproductive history, CGG repeat number, and number of AGG interruptions were known. In total, 262 women who carry a premutation were included: 94 women with 55–79 CGG repeats, 126 women with 80–100 CGG repeats, and 42 with >100 CGG repeats. There were no significant differences between groups for age at interview, race, or BMI (**Table 1**).

**Table 1:** Demographics of the study population of women with a premutation.

	55–79	80–100	>100
	Repeats	Repeats	Repeats
	(*N* = 94)	(*N* = 126)	(*N* = 42)
Age at interview [mean ± SD (range)] (*N* = 262)	45.9 ± 13.9 (18–82)	46.6 ± 11.1 (22–75)	42.2 ± 11.2 (20–60)
Race (% White) (*N* = 262)	91.5%	96.0%	95.2%
BMI (mean ± SD) (*N* = 236)	26.8 ± 6.0	25.3 ± 5.4	24.0 ± 4.5
Reproductive status		
Still cycling (*N* = 82)	37	28	17
Surgery that stopped cycles (*N* = 16)	9	6	1
Experienced amenorrhea (*N* = 164)	48	92	24

Reproductive history information was summarized using self-report questionnaires and in-depth interviews. Based on the most recent point of contact, women were classified into three groups: 82 women were still cycling at the last point of contact, 16 women had a surgery such as a hysterectomy or oophorectomy that would prevent a determination of AAA, and 164 women had had onset of menopause or secondary amenorrhea (**Table 1**).

**FIGURE 1:**
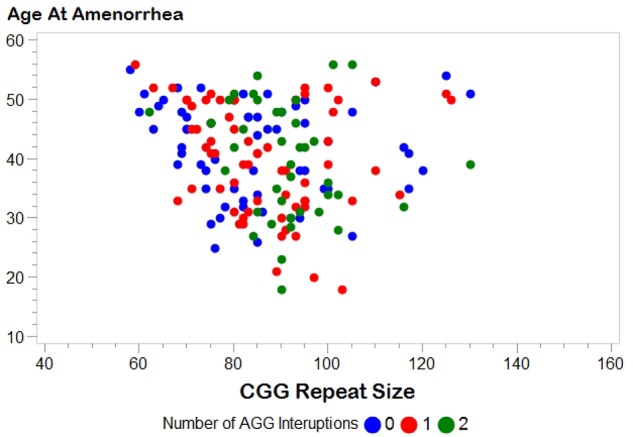
Distribution of age at amenorrhea by *FMR1* CGG repeat size. Number of AGG interruptions distinguished by color: blue, 0 AGG interruptions; red, 1 AGG interruption; green, 2 AGG interruptions.

**Figure 1** shows the relationship between AAA and repeat size and number of AGG interruptions for the 164 women with amenorrhea. In our initial analysis, we used linear regression to confirm the relationship between repeat size and AAA (**Table 2**). Model 1, based on 164 women with amenorrhea, examined both the repeat size and the quadratic transformation of repeat size to model the *U*-shaped risk curve associated with AAA. Both variables were highly significant (*p* < 0.0001), and the overall model explained 13% of the variation in AAA (*r*^2^ = 0.13). When number of AGG interruptions was added (Model 2), repeat size and the quadratic form of repeat size remained highly significant (*p* < 0.0001), but number of AGG interruptions was not significant (*p* = 0.81); thus, no additional variance in AAA was explained.

**Table 2:** Linear regression models based on women with a premutation who had experienced amenorrhea (*N* = 164).

	Repeat/3′ pure	Repeat^2^/3′ pure^2^	AGG interruptions	Overall
	β ± SE	β ± SE	β ± SE	model *r*^2^
	(*p*-value)	(*p*-value)	(*p*-value)	(*p*-value)
Model 1: AAA = repeat + repeat^2^	-1.94 ± 0.38	0.01 ± 0.00	–	0.13
	(<0.0001)	(<0.0001)		(<0.0001)
Model 2: AAA = repeat + repeat^2^ + AGG	-1.97 ± 0.40	0.01 ± 0.002	0.22 ± 0.88	0.13
	(<0.0001)	(<0.0001)	(0.8073)	(<0.0001)
Model 3: AAA = 3′ pure + 3′ pure^2^	-1.55 ± 0.32	0.01 ± 0.002	–	0.12
	(<0.0001)	(<0.0001)		(<0.0001)
Model 4: AAA = AGG+ 3′ pure + 3′ pure^2^	-1.60 ± 0.32	0.01 ± 0.002	-1.79 ± 0.90	0.13
	(<0.0001)	(<0.0001)	(0.0490)	(<0.0001)

*AAA, age at amenorrhea; β, beta coefficient of indicated variable; SE, standard error*.

In Model 3, we investigated whether using the 3′ pure repeat and its squared term explained more of the variance in AAA than the overall repeat size. We found that these variables were highly significantly associated, but did not explain additional variance in AAA. In Model 4, we added the number of AGGs to Model 3 to better define the repeat structure. Although number of AGGs was marginally significant (*p* = 0.0490), the overall variance explained by this model was similar to Model 1. In addition, the correlation for number of AGGs was negative, indicating as number of AGG interruptions increased, the AAA decreased.

These models were also run using GEE analysis to adjust for the relatedness of individuals, and the conclusions were the same (data not shown). In addition, to ensure results were not affected by memory bias of older participants, models were tested using only subjects that were less than or equal to age 60 at the time of interview. This reduced our sample size to 139 for **Table 2**, but all conclusions were the same (data not shown).

We hypothesized that the number of AGG interruptions may only impact the secondary structure up to a particular repeat size, similar to the relationship seen in the risk for instability in transmission of premutation alleles. Thus, we tested the four models presented in **Table 2** using only premutation carriers with 55–90 repeats (i.e., alleles most likely to be affected by AGG interruptions based on transmission studies). Our power was more limited because of the decrease in sample size and the reduced variability in repeat size. Nevertheless, our conclusions were consistent with the overall models presented in **Table 2**: the strongest prediction model was Model 1 using repeat size and the quadratic of repeat size (data not shown).

To include the information from the larger dataset that included women who had not experienced amenorrhea by the time of interview, we performed survival analysis (**Table 3**). In Model 1, using the repeat size and the squared term, both variables were significantly associated with AAA. In Model 2 with the number of AGG interruptions added, AGG was not a significant predictor. Similar to the linear regression model, using the 3′ pure repeat was significant, but the overall log ratio of the model was less than that for Model 1. The same findings were seen in frailty models that were adjusted for relatedness of individuals (data not shown). As before, we tested the survival models using only the premutation carriers with 55–90 repeats, and as we saw in the linear regression models, our conclusions were the same for this subset of women: the strongest model was Model 1 that included the repeat size and the quadratic form of repeat size (data not shown). We also tested these models in the subset of women that were ≤age 60 at time of interview. Our sample size for these models was reduced to 234; however, all conclusions remained the same.

**Table 3:** Survival analysis models based on all women with a premutation (*N* = 265).

	Repeat/3′ pure	Repeat^2^/3′ pure^2^	AGG interruptions	Overall
	HR; 95% CI	HR; 95% CI	HR; 95% CI	model LR
	(*p*-value)	(*p*-value)	(*p*-value)	(*p*-value)
Model 1: AAA = repeat + repeat^2^	1.22; 1.11–1.34	0.99; 0.99–0.99	–	23.51
	(<0.0001)	(<0.0001)		(<0.0001)
Model 2: AAA = repeat + repeat^2^ + AGG	1.23; 1.12–1.36	0.99; 0.99–0.99	0.94; 0.76–1.17	23.79
	(<0.0001)	(<0.0001)	(0.5945)	(<0.0001)
Model 3: AAA = 3′ pure + 3′ pure^2^	1.15; 1.07–1.25	0.99; 0.99–1.00	–	17.99
	(0.0002)	(0.0003)		(0.0001)
Model 4: AAA = AGG+ 3′ pure + 3′ pure^2^	1.15; 1.07–1.24	0.99; 0.99–0.99	1.09; 0.88–1.35	18.64
	(0.0002)	(0.0003)	(0.4187)	(0.0003)

*AAA, age at amenorrhea; HR, hazard ratio for indicated variable; CI, confidence interval; LR, Likelihood Ratio*.

## Discussion

Using data from 262 women who carry a premutation, we first confirmed the relationship between CGG repeat number in the 5′ UTR of the *FMR1* gene and AAA. Having both the repeat size and the quadratic form of repeat size in the model explained 13% of the variation in AAA among all premutation women that had gone through amenorrhea. Because we are only describing a small amount of the variation in AAA, we tested the number of AGG interruptions as a predictor variable. The number of AGG interruptions has been shown to influence the instability of the repeat in transmission, especially among smaller premutation alleles (Nolin et al., 2013, 2015). Adding the number of AGG interruptions to the model did not improve the ability to predict AAA using either linear regression analysis or survival analysis. As a secondary analysis, we tested whether our conclusions were altered if we only looked among women with 55–90 repeats. The range for this analysis was chosen because these alleles are most affected by the presence of AGG interruptions in transmission studies. Our conclusions remained the same after this analysis: the number of AGG interruptions did not add predictive power to our models for AAA.

Our results differ from those presented by Lekovich et al. (2017). They confirmed the non-linear effect of repeat size on risk for diminished ovarian reserve with their total population of 96 women with a premutation. Their population, however, only included women who were candidates for IVF and had the residual ovarian reserve for ovarian stimulation, necessarily excluding those with the most severe pathology, namely FXPOI. Working on the hypothesis that the expanded CGG repeat can form secondary mRNA hairpin structures that sequester RNA-binding proteins, they hypothesized that the AGG interruptions disrupt the hairpin formation. A premutation transcript with no AGG interruptions may have a higher level of RNA-binding protein sequestration compared to those with AGG interruptions. Among the 32 women with known AGG interruptions pattern, they reported a possible association of improved ovarian reserve for those women with AGG interruptions. However, their sample size was fairly limited, especially for women with 0 AGG interruptions (*N* = 3). It is also unknown whether their findings in women with diminished ovarian reserve are applicable in women with FXPOI, though ovarian reserve does presumably decline on a continuum (Lekovich et al., 2017).

Additional evidence for a mechanism involving inclusions formed by the protein sequestration of the CGG repeat was provided in 2013. Todd et al. (2013) demonstrated that the long CGG repeat in *FMR1* triggered a repeat-associated non-AUG-initiated (RAN) translation of a polyglycine-containing protein, FMRpolyG. The FMRpolyG was shown to accumulate in inclusions in both model systems and in brains of FXTAS patients (Todd et al., 2013). Inclusion bodies have also been implicated in FXPOI. Chang et al. (2011) reported inclusions in the ovarian stromal cells of five women with a premutation and not in controls. Based on remaining tissue from one premutation carrier, Buijsen et al. (2016) detected FMRpolyG in the inclusions of the stromal cells, but not in the oocytes, granulosa cells, or theca cells. Similarly, inclusions with FMRpolyG were detected in the stromal cells of their premutation mouse model, and the greatest frequency of inclusions was seen in the older mice (Buijsen et al., 2016).

Both the sequestration and RAN translation models are based on the formation of a hairpin structure as the primary trigger for the downstream consequences. Thus, ruling out AGG interruptions as a predictive variable may not implicate one model over the other. The presence of AGG interruptions was not found to affect mRNA levels (Yrigollen et al., 2011) or FMRP translation (Ludwig et al., 2009). Studies that show an effect on secondary structure with AGG interruptions have focused on alleles that are smaller than the premutation size range (Napierala et al., 2005; Jarem et al., 2010). Perhaps the underlying etiology among permutations is more tolerant to the stability or the actual form of the secondary structure that is associated with number of AGG interruptions. At least for the initiation of RAN translation, the presence of AGGs does not change the composition of FMRpolyG because both the GGA and GGC codon code for a glycine amino acid. Thus, a disruption of the CGG repeat would not change the potential polyG reading frame.

The question remains as to why there is a non-linear relationship with CGG repeat size and AAA. With respect to RAN translation model, Sellier et al. (2017) found that the FMRpolyG was hardly detectable below 70 repeats. This could explain the increased risk for FXPOI starting at about 70 repeats. In addition, as the repeat increases, the levels of FMRP are reduced (Tassone et al., 2000; Kenneson et al., 2001). Perhaps the reduction in FMRP or its downstream effects reduce the risk of FXPOI for women with larger premutation alleles.

There are several limitations to the work presented here. First, a woman who experiences early ovarian insufficiency could potentially be more willing to participate in research. This could potentially inflate our proportion of women with FXPOI; however, this would not affect the question of the association of AGG interruptions because we did not select participants based on repeat structure. Second, women in families with a history of FXS are more likely to be recruited for our research. Thus, we are more likely to identify women with unstable alleles (i.e., smaller alleles with 0 AGGs or larger alleles with any number of AGGs). **Figure 1** shows a fairly similar distribution between CGG repeat size and AAA for each of the AGG groups, but we do not know for certain if this is representative of the premutation alleles seen in the general population. Finally, age at secondary amenorrhea is primarily based on self-report. Having interviews with a reproductive endocrinologist on a subset strengthens the dataset; however, we did not confirm any of the data with medical records. Irrespective, we do not expect that any noise associated with self-report will vary by repeat structure.

## Conclusion

In summary, among our population of 262 women with a premutation, we were not able to identify an association with AAA and number of AGG interruptions once models were adjusted for repeat size. Importantly, our conclusions remained consistent across the various models that were tested. This observation provides one more clue to the underlying etiology of the risk for FXPOI. Because only 13% of the variation in AAA among premutation carriers is explained by repeat size, it is clear that further research is needed to identify other predictive factors, both genetic and environmental, that affect the risk for the onset of FXPOI.

## Author Contributions

EA performed the data analysis and manuscript preparation. AG, NT, WH, AA, and SN performed the lab work. KC, LS, and HH participated in subject recruitment. AG, KC, HH, SN, and SS contributed to manuscript preparation.

## Conflict of Interest Statement

The authors declare that the research was conducted in the absence of any commercial or financial relationships that could be construed as a potential conflict of interest.

## Supplementary Material

TABLE S1 Raw data for each subject included in analyses. Age at Interview is age of most recent reproductive questionnaire or interview. Age at Amenorrhea is the self-reported age of last menstrual period for women that have gone more than 4 months without a period. *FMR1* allele 1 and allele 2 are the number of CGG repeats determined by genotyping. Number of AGGs is the number of AGG interruptions seen in the premutation allele.
